# Genetic Characterization of a Recombinant Myxoma Virus in the Iberian Hare (*Lepus granatensis*)

**DOI:** 10.3390/v11060530

**Published:** 2019-06-07

**Authors:** Ana Águeda-Pinto, Ana Lemos de Matos, Mário Abrantes, Simona Kraberger, Maria A. Risalde, Christian Gortázar, Grant McFadden, Arvind Varsani, Pedro J. Esteves

**Affiliations:** 1 CIBIO/InBio—Centro de Investigação em Biodiversidade e Recursos Genéticos, Universidade do Porto, Campus Agrário de Vairão, 4485-661 Vairão, Portugal; anaagueda@cibio.up.pt; 2 Departamento de Biologia, Faculdade de Ciências, Universidade do Porto, 4169-007 Porto, Portugal; 3 Center for Immunotherapy, Vaccines, and Virotherapy (CIVV), The Biodesign Institute, Arizona State University, Tempe, AZ 85287, USA; alemosde@asu.edu (A.L.d.M.); mruidias@asu.edu (M.A.); grantmcf@asu.edu (G.M.); 4 The Biodesign Center for Fundamental and Applied Microbiomics, Center for Evolution and Medicine and School of Life sciences, Arizona State University, Tempe, AZ 85287, USA; simona.kraberger@asu.edu (S.K.); arvind.varsani@asu.edu (A.V.); 5 Dpto. de Anatomía y Anatomía Patológica Comparadas, Universidad de Córdoba, Agrifood Excellence International Campus (ceiA3), 14071 Córdoba, Spain; MariaA.Risalde@uclm.es; 6 Instituto de Investigación en Recursos Cinegéticos IREC-CSIC-UCLM-JCCM, Ronda de Toledo, 28005 Ciudad Real, Spain; Christian.Gortazar@uclm.es; 7 Structural Biology Research Unit, Department of Clinical Laboratory Sciences, University of Cape Town, Cape Town 7701, South Africa; 8 CITS—Centro de Investigação em Tecnologias da Saúde, IPSN, CESPU, 4585-116 Gandra, Portugal

**Keywords:** poxvirus, Myxoma virus, recombinant virus, *Lepus granatensis*

## Abstract

Myxomatosis is a lethal disease in wild European and domestic rabbits (*Oryctolagus cuniculus*), which is caused by a Myxoma virus (MYXV) infection—a leporipoxvirus that is found naturally in some *Sylvilagus* rabbit species in South America and California. The introduction of MYXV into feral European rabbit populations of Australia and Europe, in the early 1950s, demonstrated the best-documented field example of host–virus coevolution, following a cross-species transmission. Recently, a new cross-species jump of MYXV has been suggested in both Great Britain and Spain, where European brown hares (*Lepus europaeus*) and Iberian hares (*Lepus granatensis*) were found dead with lesions consistent with those observed in myxomatosis. To investigate the possibility of a new cross-species transmission event by MYXV, tissue samples collected from a wild Iberian hare found dead in Spain (Toledo region) were analyzed and deep sequenced. Our results reported a new MYXV isolate (MYXV Toledo) in the tissues of this species. The genome of this new virus was found to encode three disruptive genes (*M009L*, *M036L*, and *M152R*) and a novel ~2.8 kb recombinant region, which resulted from an insertion of four novel poxviral genes towards the 3’ end of the negative strand of its genome. From the open reading frames inserted into the MYXV Toledo virus, a new orthologue of a poxvirus host range gene family member was identified, which was related to the MYXV gene *M064R*. Overall, we confirmed the identity of a new MYXV isolate in Iberian hares, which, we hypothesized, was able to more effectively counteract the host defenses in hares and start an infectious process in this new host.

## 1. Introduction

Myxoma virus (MYXV), a poxvirus belonging to the *Leporipoxvirus* genus, is the etiological agent of myxomatosis, which is a highly lethal viral disease in wild and domestic European rabbits (*Oryctolagus cuniculus*) [1]. The classical form of the disease is characterized by a systemic spread of the virus, overwhelming the immune system, and the development of secondary skin lesions called “myxomas” [2,3]. Mortality rate varies between 20%–100%, according to the grade of virulence of the MYXV strain [3]. The virus has its natural host in the South American tapeti, or forest rabbit (*Sylvilagus brasiliensis*), where it causes an innocuous and localized cutaneous fibroma, at the inoculation site [2]. Poxviruses related to MYXV are found in other *Sylvilagus* species in North America—the Californian MYXV strains, for which the natural host is *Sylvilagus bachmani* (brush rabbit), and the rabbit fibroma virus (RFV) found in *Sylvilagus floridanus* (eastern cottontail) [2,4]. The MYXV does not seem to cause significant clinical diseases in the natural *Sylvilagus* hosts, though being highly pathogenic to the naive *Oryctolagus* host makes it a classic example of a pathogen that is highly virulent in a new host species with no evolutionary history of adaptation to that pathogen.

In 1950, with the urge of controlling the infesting population of European rabbits in Australia, a MYXV strain originally isolated in Brazil (standard laboratory strain [SLS]) was used as a biological agent [1]. The release of a different Brazilian isolate of MYXV in 1952 France (Lausanne [Lau] strain), resulted in the establishment and spread of the MYXV in Europe, including the United Kingdom (UK) [5]. After an initial massive reduction of the wild rabbit populations (>99%) in both continents, a substantial decline in the case fatality rates occurred as a result of the natural selection for slightly attenuated viruses, but also due to an increased resistance to myxomatosis in the rabbit populations [4,6,7]. It has been recently shown that the convergent phenotype of viral resistance observed in Australia, France, and UK rabbit populations was followed by a strong pattern of parallel evolution, a consequence of selection acting on the standard genetic variation that was present in the ancestral rabbit populations in continental Europe [8].

The susceptibility of other leporids species to MYXV has been tested in controlled experiments, while evidence of myxomatosis in wild leporid populations have been seldom reported. Using a California MYXV strain, four different North American *Sylvilagus* species (*S. audubonii*, *S. floridanus*, *S. idahoensis* [now *Brachylagus idahoensis*], and *S. nuttallii*) developed tumors, following mosquito transfers, but these failed to be mosquito-infective lesions [9]. Three of these *Sylvilagus* species (*S. audubonii*, *S. floridanus*, and *S. nuttallii*) when infected with the Brazilian Lau strain also developed prominent tumors, however, in this case the South American strain produced mosquito-infective lesions [10]. On the other hand, black-tailed jackrabbits (*Lepus californicus*) inoculated with Californian MYXV did not form tumors [9]. In wild populations of the European hare (*Lepus europaeus*), cases of myxomatosis have been reported sporadically and in small numbers. In the past, the confirmation of the disease arose from injecting rabbits with tissues from dead hares and replicating its typical clinical symptoms [11]. Most recently, in 2014, for the first time, a case of myxomatosis was confirmed in a European brown hare in Great Britain, using electron microscopy and PCR of a skin lesion [12].

Recently, in late summer–fall of 2018, the first cases of myxomatosis were reported in the wild Spanish Iberian hare (*Lepus granatensis*) populations, mainly in the Andalusia and Castilla-La Mancha regions. The Spanish Ministry of Agriculture, Fisheries, and Food, and the Institute for Game and Wildlife Research identified what appeared to be a cross-species transmission into a new leporid species. Iberian hares were found in a moribund state, with signs of blindness, weakness, and disorientation, and were consequently analyzed in different laboratories. Here, using culturing and deep sequencing, we genetically characterized, for the first time, a recombinant MYXV isolated from an Iberian hare carcass exhibiting classical symptoms of myxomatosis collected in the Toledo province of Spain, during the 2018 outbreak (referred to as MYXV Toledo).

## 2. Methods

### 2.1. Sampling and Pathology

**Figure 1:**
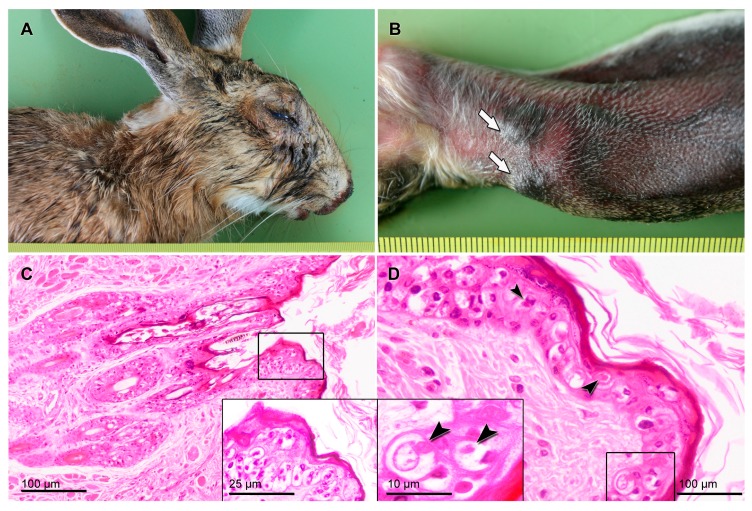
Iberian hare with myxomatosis-compatible lesions. (**A**) Blepharitis and conjunctivitis with seropurulent discharge. (**B**) Myxomas at the base of the left ear (arrows). (**C**) Severe acanthosis of the eyelid skin, with hyperkeratosis. (**D**) Ballooning degeneration of the epidermal cells, and intracytoplasmic eosinophilic inclusion bodies in the eyelid skin (arrowheads).

An adult Iberian hare (*L. granatensis*) female, was found dead on 21st of August 2018 in La Villa de Don Fabrique municipality in the Toledo province of Spain. The hare manifested lesions compatible with myxomatosis in European rabbits (Figure 1) and was completely emaciated (kidney fat index = 0). On arrival at the laboratory, duplicate samples (4 mm diameter) were taken from the eyelids, ears, and vulva, and stored in RNAlater, without preservatives, at −80 °C. For the histopathological study, representative samples of the main organs and tissues were fixed in 10% buffered formalin for 48–72 h, at 22 ± 2 °C, and then dehydrated in a graded series of ethanol, immersed in xylol, and embedded in paraffin wax, using an automatic processor. Sections were cut at 4 µm and stained with hematoxylin and eosin (H-and-E), following standard procedures.

### 2.2. Cell Lines

European rabbit RK13 kidney epithelial cells (Millipore Sigma, Massachusetts, MA, USA) were maintained in Dulbecco’s modified Eagle medium (HyClone, Utah, UT, USA), supplemented with 10% fetal bovine serum (FBS), 2 mM L-glutamine, and 100 U/mL of penicillin/streptomycin. The Cells were maintained at 37 °C in a humidified 5% CO_2_ incubator.

#### 2.2.1. Isolation, Replication, and Purification of the New Myxoma Virus (MYXV Toledo)

Samples from the lesions of the eyelid of an Iberian hare (*L. granatensis*) specimen were manually homogenized. A small volume (5–10 μL) of the processed tissues was used to inoculate confluent RK13 cells monolayers in a 6-well plate, and was allowed to incubate at 37 °C. Two days after infection, distinctive MYXV foci were visualized using a Leica DMI6000 B inverted microscope (Supplementary Figure S1). To proceed with the virus isolation, the infected cells were harvested, thrice freeze–thawed at −80 °C and 37 °C, and sonicated for one minute, to release the viruses from the infected cells. The virus was inoculated back onto a confluent RK13 cells monolayer in a 150 mm dish and incubated at 37 °C for 48 h. The cells were collected to perform a serial dilution and the one with the best individualized foci (dilution 10^−5^) was used for inoculating a new 150 mm dish. After 2 days of infection, a last round of cell harvest, freeze–thaw cycles, and sonication was done, before proceeding to virus amplification into twenty 150 mm dishes. Purification of the virus through a 36% sucrose cushion was performed, as described before [13]. Titration of the number of plaque forming units of the virus was determined by crystal violet foci staining of the infected RK13 cell monolayers, while the total number of viral particles was counted using the NanoSight NS300 instrument (Malvern Panalytical, Malvern, UK).

#### 2.2.2. Viral Nucleic Acid Extraction, Illumina Sequencing, and De novo Assembly of the Genome

Total viral nucleic acid was extracted from 200 µL of the viral preparation, using a phenol–chloroform extraction protocol, as previously described [14]. The viral DNA was used to generate a 2 × 100 bp Illumina sequencing library and this was sequenced on a Illumina HiSeq4000 (Illumina, USA) at Macrogen Inc. (Korea). The paired-end raw reads (40,730,938 reads) were de novo assembled using metaSPAdes v3.12.0 [15] with kmer of 33, 55, and 77. The de novo assembled contigs were then assembled into a genome length contig, using MYXV-Lau (GenBank accession # MK836424) as a scaffold, primarily to resolve the terminals’ redundancy. The quality of the final assembly was verified by mapping the raw reads back to the genome, using BBMap [16] (Supplementary Figure S2).

### 2.3. Genome Analysis

All MYXV and poxvirus RefSeqs were downloaded from GenBank on 9 April, 2019. Global alignments of the MYXV with the genome determined in this study were carried out using MAFFT [17]. ORFs in the genome were determined with ORFfinder (https://www.ncbi.nlm.nih.gov/orffinder/), coupled with a local MYXV ORF database generated from the MYXV genomes. ORFs that did not have any similarity to MYXV ORFs were analyzed using BLASTn and BLASTx sequence queries [18]. All pairwise identities (nucleotide and protein) were calculated using SDV v1.2 [19].

Nucleotide sequence and protein sequence aligns were undertaken using MAFFT [20]. The nucleotides alignments of the genomes and the recombinant gene “cassette”-like sequences were used to infer maximum likelihood phylogenetic trees using PHYML 3.0 [21], with the substitution model GTR+G+I. Amino acid alignments of the newly derived poxvirus virion protein, thymidine kinase, host range protein, and the poly(A) polymerase subunit were used to infer maximum likelihood phylogenetic trees using PHYML 3.0 [21], with substitution models JTT+G, WAG+G+F, JTT+G+F, and JTT+G+F, respectively; determined using ProtTest [22]. Branches with an aLRT support <0.8 were collapsed using TreeGraph2 [23].

## 3. Results and Discussion

The natural host for MYXV is the South American tapeti (South American strains) [2,4]. As expected from the predictions of the long-term virus/host co-evolution, MYXV strains are highly adapted to their natural hosts, causing only benign cutaneous fibromas [4]. However, when another susceptible host becomes available to the virus transmission system, in this case the European rabbit (*Oryctolagus cuniculis*), a successful cross-species transmission can occur. Indeed, when MYXV first entered the European rabbit host, it was immediately pathogenic and caused close to 100% mortality. After the use of MYXV in the 1950s to control feral rabbit populations in Australia and Europe, rapid co-evolutionary changes occurred in both the rabbit host and the virus, due to an increased resistance in rabbit populations and the appearance of less virulent virus strains [8,24]. In 2014, a study reported the presence of a myxomatosis-like disease in the European brown hare (*Lepus europaeus*) [12]. However, a MYXV virus capable of infecting hares has not been previously genetically characterized. More recently, reports of abnormal mortalities in Iberian hares were described in the Spanish regions of Andalucía, Castilla-La Mancha, Extremadura, Madrid, and Murcia. The animals found in the hunting grounds presented with inflammation of the eyelids, conjunctivitis, and also inflammation of the perianal area, symptoms consistent with classic rabbit myxomatosis.

In this study, a new MYXV virus (MYXV Toledo) was isolated and sequenced from an Iberian hare found in the Toledo province, which presented the classical lesions of myxomatosis, including a bilateral blepharitis, conjunctivitis and a swollen vulvar and anal region (Figure 1A,B). The basal third of the left ear presented two myxoma-like lesions of a 5 mm diameter (Figure 1B). Moreover, epistaxis and strong congestion of the trachea were observed, whereas the lung was swollen, and presented few petechial hemorrhages. Histopathology analysis of the eyelid skin revealed the typical proliferation and ballooning degeneration of the epidermal cells, containing single, large, rare, intracytoplasmic, round, and eosinophilic inclusion bodies (Figure 1C). In this tissue, a severe acanthosis with erosion and ulceration was observed. Blepharoconjunctivitis lesions were also associated with an inflammatory cell response in the underlying dermis, with infiltration of large macrophage-like cells, diffuse edema, and fibrin deposition (Figure 1D). In the lung, mild congestion, alveolar edema, and hemorrhages were observed. These vascular lesions were also recorded in the liver and kidneys.

### 3.1. Comparison of MYXV Lausanne variant with the Newly Discovered MYXV Toledo Variant and RFV Kasza

**Figure 2:**
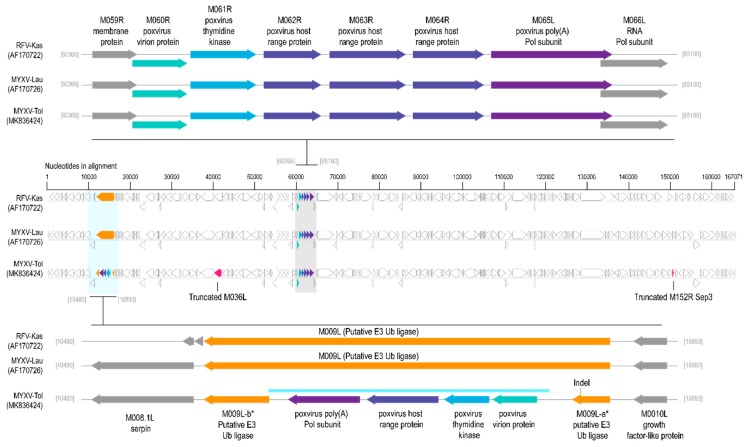
Representation of the aligned genome organization of both RFV-Kas (AF170722), MYXV-Lau (AF170726), and Myxoma virus-Toledo (MYXV-Tol) (MK836424). Blue ORF illustrations represent truncated genes; purple shows the location of *M060R*, *M061R*, *M062R*, *M063R*, *M064R*, and *M065R* genes in both MYXV virus; orange shows the *M009L* gene (intact in RFV-Kas, MYXV-Lau, and disrupted in MYXV-Tol) and shades of blue represent the new gene “cassette” identified in MYXV-Tol, which is highly likely derived from a recombinant event with an unsampled poxvirus.

From the collected samples, a new MYXV was isolated which we have named as ‘MYXV Toledo (MYXV-Tol)’. The de novo assembled genome was 164,579 bps (Supplementary Figure S2). This genome was aligned to MYXV-Lau (GenBank accession # AF170726) and RFV-Kas (GenBank accession # AF170722), for a preliminary analysis (Figure 2). The MYXV-Tol genome (GenBank accession # MK836424) was found to be ~2,800 bp longer than the one reported for the MYXV-Lau strain (161,777 bp) [25]. Based on the published genome sequence, the MYXV-Lau strain has a total of 171 genes (12 of which are duplicated in the terminal inverted repeats (TIRs) regions) [3,25] and can be divided into three regions—the terminals, 14.1 kb extending from the left TIR (*M0005.1L* to *M011L*), and 23.1 kb extending from the right TIR (*M143R* to *M000.5L*), which mostly contain genes involved in the MYXV virulence and host subversion, while the central 124.5 kb region (*M012L* to *M142R*) includes a mixture of virulence genes and essential viral genes conserved across all poxviruses [2,26]. Around ~99 % of the encoded gene products of MYXV-Tol were identical to those of MYXV-Lau, with the exception of the ORFs *M009L*, *M152R*, and *M036L*. Phylogenetically, MYXV-Tol clusters with the majority of the MYXV genomes (Supplementary Figure S3; Supplementary Data 2). Furthermore, we identified a novel insertion of ~ 2800 bp within the *M009L* gene that spans the 12,236 to 15,082 bp region (i.e., within the 10,480–16,893 nts alignment position in Supplementary Data 1) at the left end of the MYXV-Tol genome (Figure 2).

### 3.2. Viral Genes Disrupted in the New MYXV-Tol Isolate

As previously reported for the MYXV isolates from feral rabbits in Australia and Great Britain, single or multiple indels that result in the disruption of ORFs are relatively common [27,28,29]. In the Lausanne strain, *M009L* encoded a putative E3 ubiquitin (Ub) ligase of 509 aa with a N-terminal BTB-BACK domain, followed by 4 Kelch motifs [30]. Our genomic analysis revealed that ORF *M009L* of MYXV-Tol was disrupted by an insertion of four nucleotides (+TATA, at position 15,586 bp), causing a frameshift mutation. This indel resulted in a smaller truncated M009L predicted protein of 148 aa. Several reports have shown that this same gene was also disrupted in multiple Australian MYXV strains [28], as well as in the Californian MSW strain [16], which suggest that the disruption of this gene does not abrogate MYXV survival in the wild. Four additional nucleotides were also found in the *M036L* gene (+TTTT, position 42,007 bp), thereby creating a premature stop codon in the frame, within this gene. M036L is an orthologue of the O1 protein that is found in the orthopoxvirus vaccinia virus (VACV) [29]. However, the function of M036L in the MYXV virus has not been reported. A previous study has shown that certain MYXV field isolates carry a deletion of 89 nt in this gene [31]. However, this indel appeared to have no major effects in the survival and spread of MYXV in rabbits [31]. In the MYXV-Lau, ORF M152R encodes a serine proteinase inhibitor (Serp3) of 266 aa [32]. In the MYXV-Tol virus, this gene was disrupted as a result of an insertion of a single nucleotide (+C, at position 150,688 bp), resulting in the appearance of an early stop codon. The exact biological function of Serp3 in MYXV is not known. To date, two other serpins have been identified in MYXV, Serp1 and Serp2 [33], both of which are implicated in the modulation of host inflammatory responses [34,35,36]. Phenotypically, the deletion of specific host range proteins inevitably results in the reduced ability of the resulting virus to infect cells or tissues of species, for which the parental virus was adapted. For this reason, we consider it to be less likely that the truncation of *M152R* contributes to the observed virulence of MYXV-Tol in Iberian hares.

### 3.3. Analyses of the New Recombinant Region of the MYXV-Tol Isolate

**Figure 3:**
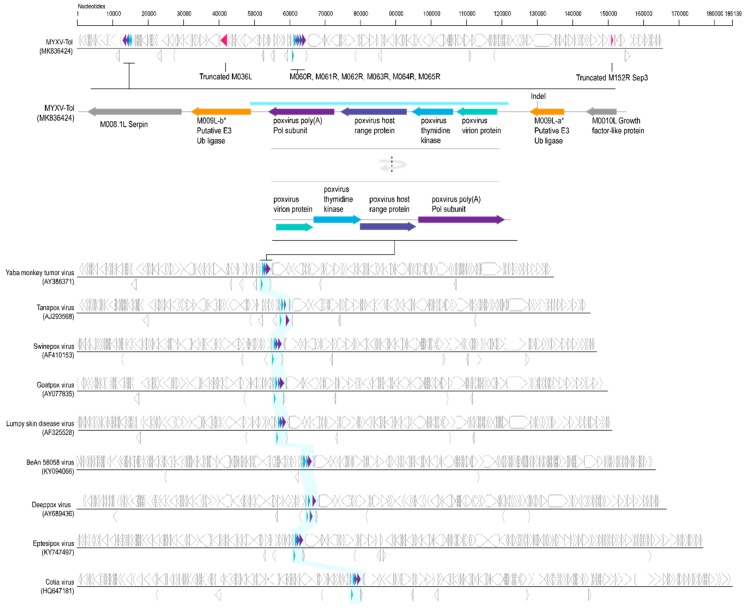
Illustration of the “four gene cassette” sequences identified in the genomes of the representative capripoxviruses, cervidpoxviruses, suipoxviruses, yatapoxviruses, and three unclassified poxviruses (BeAn 58058 virus, cotia virus and eptesipoxvirus) that share similarity to the one found encoded on the negative strand of MYXV-Tol genome sequence.

**Figure 4:**
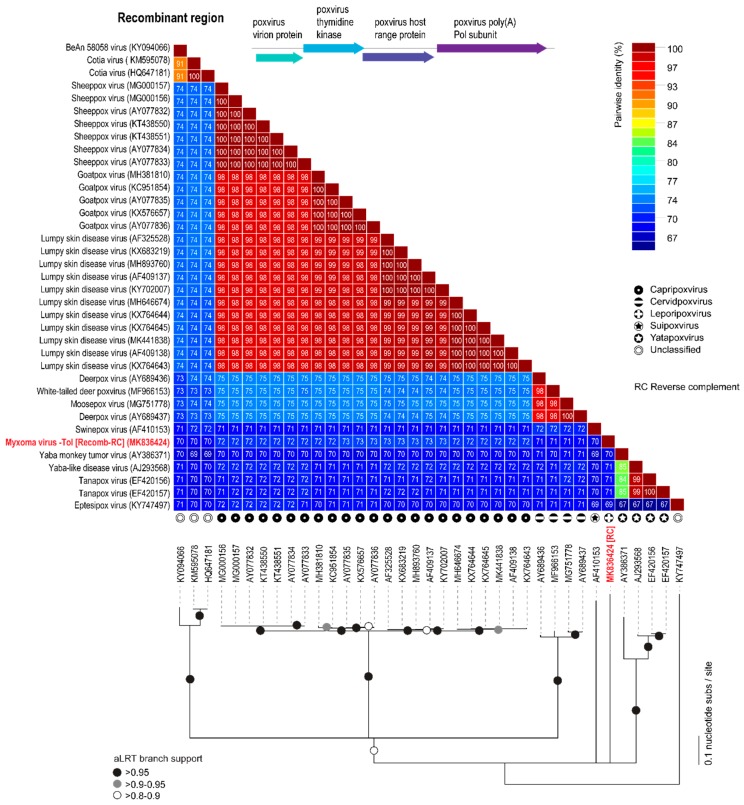
Pairwise nucleotide identity matrix (upper image) and maximum-likelihood phylogenetic tree (model GTR+G+I) showing the relationships of the MYXV-Tol recombinant “four gene cassette” to similar sequences in the genomes of capripoxviruses, cervidpoxviruses, suipoxviruses, yatapoxviruses, and three unclassified poxviruses (BeAn 58058 virus, cotia virus, and eptesipoxvirus). Branches with aLRT support of 0.95 are indicated by black circles whereas branches exhibiting 0.9–0.95 and 0.8–0.9 aLRT supports are indicated by grey and white circles, respectively.

Analyses of the MYXV-Tol genome sequence revealed an insertion of ~2800 bp in the left side of the genome (Figure 2). This new recombinant region encodes at least four genes that are predicted to encode four viral proteins that are homologous, but not identical, to the poxvirus gene families exemplified by the *M060R*, *M061R*, *M064R*, and *M065R* genes from MYXV. We exploited the sequence similarity searches to predict the functions of these new MYXV-Tol proteins. According to the obtained results, the recombinant region encodes a known virion protein (rPox-virion protein), followed by a thymidine kinase (Recombinant pox virus thymidine kinase; rPox-thymidine kinase), a C7L-like host range protein (rPox-host range protein), and a poly A polymerase subunit (rPox-poly(A) Pol subunit) (Figure 2). In the MYXV-Lau genome, the region that spans the locus at ~57,500 bp includes a set of six genes that are present in all MYXV (*M060R* to *M065R*) [25,37]. The predicted functions for the proteins found in the recombinant region are in accordance with those found in the ~57,500 bp region of other MYXV virus [37]. However, it should be noted that the *M062R* and the *M063R* genes that are present in all MYXV virus are not present in the new recombinant insertion region at the left end (encoded on the minus strand) of MYXV-Tol (Figure 2). A BLASTn base coupled with complete genome searches of all poxviruses for the complete recombinant region, with four new gene “cassette”, revealed that this virus gene arrangement was only found in the genomes (encoded on the positive strand) of capripoxviruses, cervidpoxviruses, suipoxviruses, yatapoxviruses, and three unclassified poxviruses (BeAn 58058 virus, cotia virus, and eptesipoxvirus; Figure 3) sharing 69–73% of nucleotide identity (Figure 4). These results suggest that the recombinant region was derived from a new, still-unreported or unsampled poxvirus, which shares a common ancestral origin with capripoxviruses, cervidpoxviruses, suipoxviruses, yatapoxviruses, and three unclassified poxviruses (BeAn 58058 virus, cotia virus, and eptesipoxvirus).

Occurrences of recombination between leporipoxviruses have been described before. In fact, it has been established that the malignant rabbit fibroma virus (RFV) is a result of a recombination event between two other leporipoxviruses, the Shope rabbit fibroma virus (SRFV), and MYXV [24,25]. The recombinant MRV is capable of immunosuppression and fatal malignancy in a broader host range, unlike the case of SFV but similar to that of MYXV [26,27,28,29].

**Figure 5:**
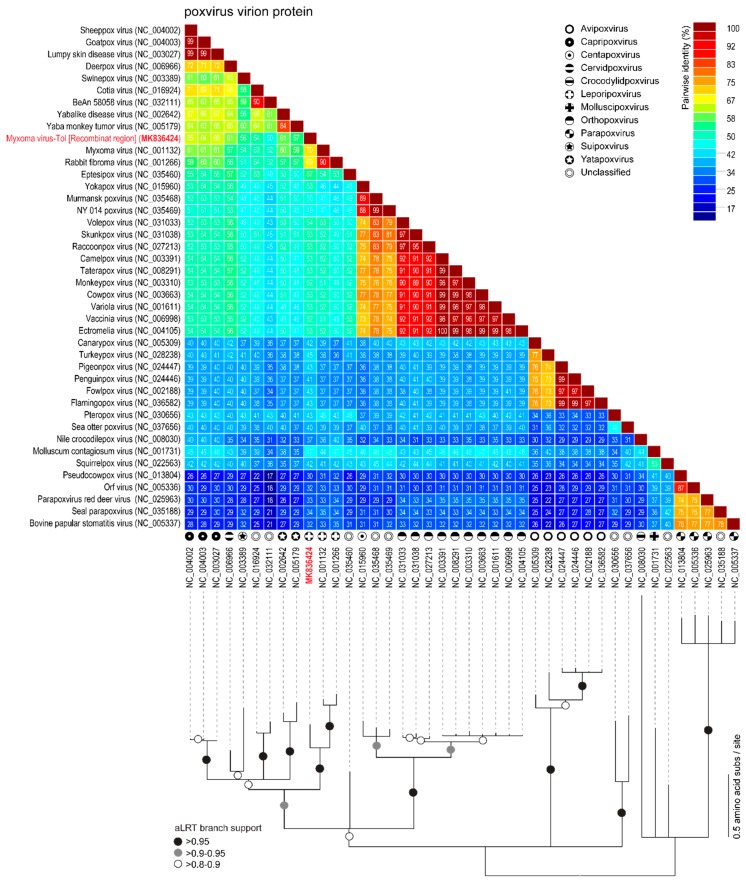
Pairwise amino acid identity matrix (upper image) and maximum-likelihood phylogenetic tree (model JTT+G), showing the relationships of the rPox-virion protein (highlighted in red) found in the recombinant region of MYXV-Tol and its homologous proteins, found in representative sequences (NCBI RefSeq) of poxvirus. Branches with aLRT support of 0.95 are indicated by black circles whereas branches exhibiting 0.9–0.95 and 0.8–0.9 are indicated by grey and white circles, respectively.

**Figure 6:**
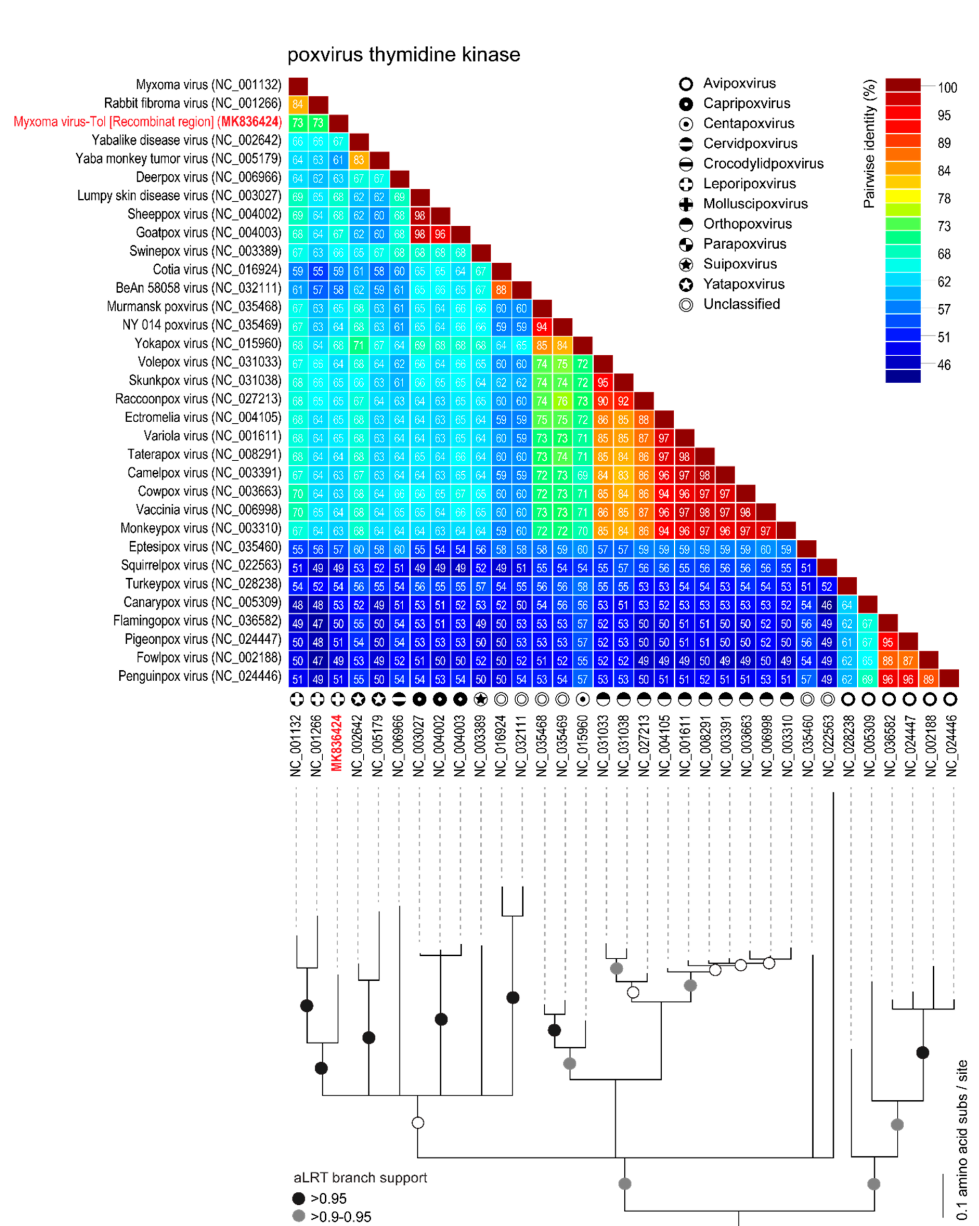
Pairwise amino acid identity matrix (upper image) and maximum-likelihood phylogenetic tree (model WAG+G+F), showing the relationships of the rPox-thymidine kinase (highlighted in red) found in the recombinant region of MYXV-Tol and its homologous proteins found in representative sequences (NCBI RefSeq) of poxvirus. Branches with aLRT support 0.95 are indicated with black circles, whereas branches exhibiting 0.9–0.95 and 0.8–0.9 are indicated with grey and white circles, respectively.

**Figure 7:**
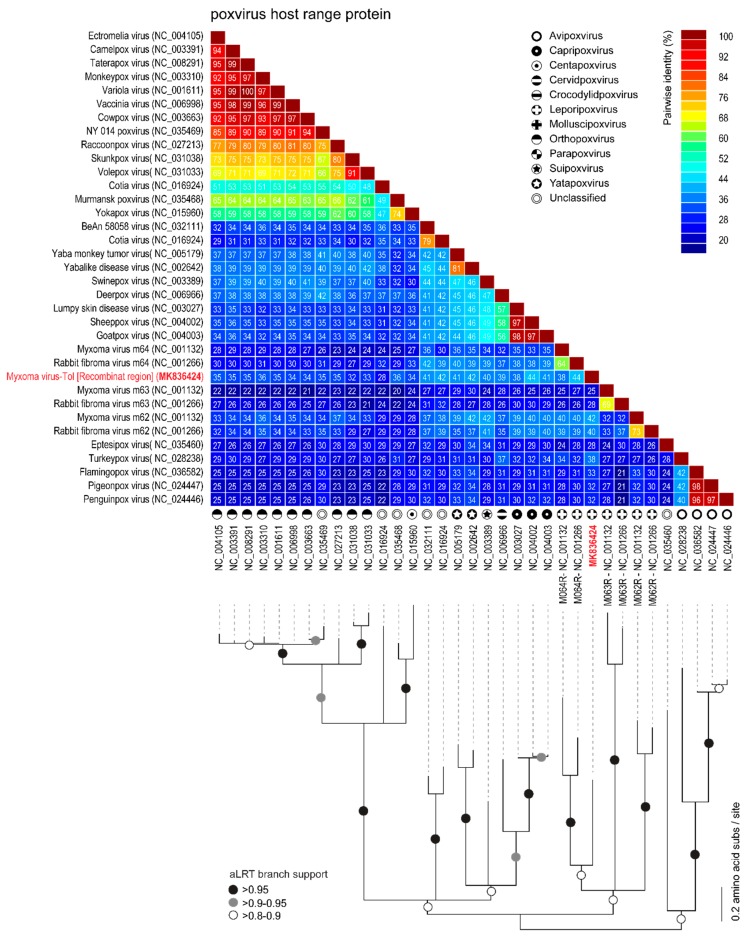
Pairwise amino acid identity matrix (upper image) and maximum-likelihood phylogenetic tree (JTT+G+F) showing the relationships of the rPox-host range protein (highlighted in red) found in the recombinant region of MYXV-Tol and its homologous proteins found in representative sequences (NCBI RefSeq) of poxvirus. Branches with aLRT support 0.95 are indicated by black circles whereas branches exhibiting 0.9–0.95 and 0.8–0.9 are indicated by grey and white circles, respectively.

**Figure 8:**
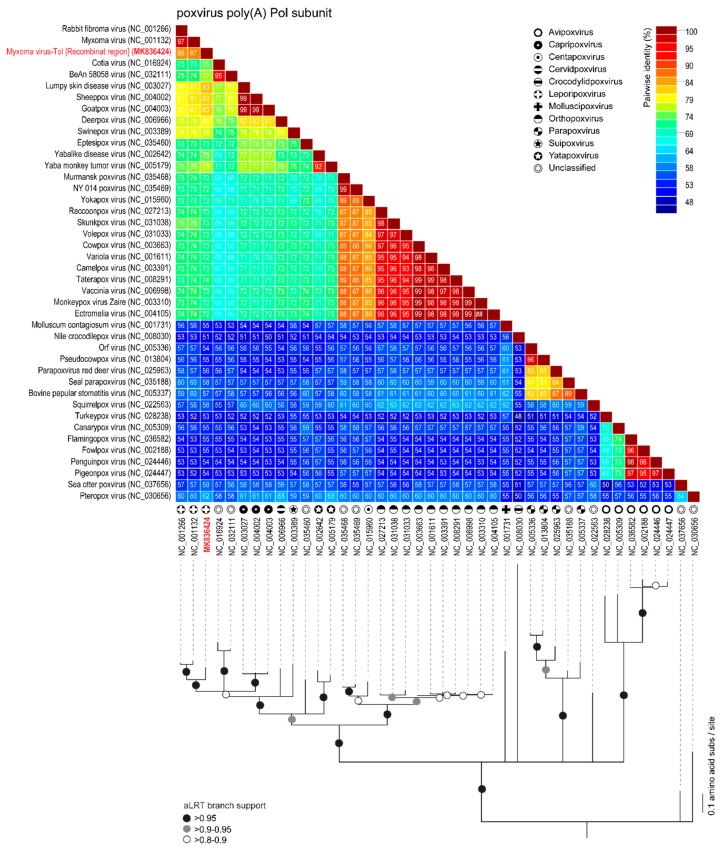
Pairwise amino acid identity matrix (upper image) and maximum-likelihood phylogenetic tree (model JTT+G+F) showing the relationships of the rPox-poly(A) Pol subunit (highlighted in red), found in the recombinant region of MYXV-Tol, and its homologous proteins, found in the representative sequences (NCBI RefSeq) of poxvirus. Branches with aLRT support 0.95 are indicated by black circles whereas branches exhibiting 0.9–0.95 and 0.8–0.9 are indicated by grey and white circles, respectively.

The rPox-thymidine kinase predicted protein sequence was found to share ~70% of its identity with its homologous protein from leporipoxviruses and 60–65% of identity with that of capripoxviruses and cervidpoxviruses (Figure 5). The rPox-virion protein predicted protein sequence was found to share 73% amino acid identity with those of leporipoxviruses and 63–70% with those of centapoxviruses, capripoxviruses, and orthopoxviruses (Figure 6). rPox-poly(A) Pol subunit shares the highest amino acid pairwise identity (80–86%) with those from capripoxviruses, cervidpoxviruses, and leporipoxviruses (Figure 7). On the other hand, the newly identified rPox-host range protein of MYXV-Tol was the least conserved among the proteins found in the recombinant region, sharing only 35–40% amino acid identity with the M064R protein family of centapoxviruses, cervidpoxviruses, and leporipoxviruses (Figure 8). Moreover, it should be noticed that the new rPox-host range protein was also found to share ~40% identity to the M062R protein found in MYXV strains and RFV and ~28% amino acid pairwise identity to the M063R protein, also found in MYXV and SRFV. Although the proteins found in the new recombinant region of MYXV-Tol shared higher pairwise identity to their homologous versions found in leporipoxviruses, it should be noted that in most cases, a small difference (~5% pairwise identity) segregated them from, for example, centapoxviruses and cervidpoxviruses. Moreover, and as mentioned before, the new recombinant region only presented one member of the C7L-like host range gene superfamily. In fact, leporipoxviruses constituted a unique example in the evolution of this gene family, since they encoded three related C7L-like gene members in tandem, *M062R*, *M063R*, and *M064R* [30]. It has been suggested that the emergence of these three C7L-like gene copies in MYXV arose after two events of gene duplication [30]. In our results, we reported that the new recombinant insertion region of MYXV-Tol only contained one predicted host range protein (Figure 2 and Figure 3), which reinforced our hypothesis that this new gene insertion region found at the left end of the MYXV-Tol genome was probably not a result of a recombinant event between two leporipoxviruses, but rather between MYXV and a still-unidentified poxvirus.

## 4. Concluding Remarks

Other than the disruption of *M009L*, *M036L*, and *M152R*, the MYXV-Tol was found to have a full complement of genes present in other MYXV isolates and strains. So the question arises as to how MYXV-Tol (with a new recombination insertion region derived from an unreported poxvirus with an common origin to capripoxviruses, cervidpoxviruses, suipoxviruses, yatapoxviruses and three unclassified poxviruses—BeAn 58058 virus, cotia virus and eptesipoxvirus), likely became pathogenic in Iberian hares. Examination was carried out on the four new poxvirus genes found in the recombinant “cassette” at the left end of the MYXV-Tol genome (encoded on the negative strand), which might induce factor(s) that mediate host range or immunosuppression in hares, allowing an increased infection and propagation of this new virus in hares. Regarding virulence in hares, it was likely that the acquisition of new genes involved in immunosuppression or host-range functions in specific cell types might have a preponderant role in this apparent species leaping of MYXV-Tol [38]. From the four new genes present in the recombinant insertion region, rPoxhost range protein is the clear candidate that suggests a possible function in the novel host interactions for this new recombinant poxvirus. As mentioned before, in the Lausanne strain, M064R belong to the C7L-like host range factor superfamily that are known to be important for MYXV pathogenesis [39,40,41]. However, since this *M064R*-like gene of MYXV-Tol shares relatively low similarity (~40%) to its orthologous proteins found in other leporipoxviruses, it is likely that this new protein has acquired new roles, perhaps reflected by alternative host targets, compared to those in the known MYXV strains. Host range proteins are defined as a group of virus-produced proteins that important for the capacity of the virus to infect cells or tissues of certain species [42]. The capacity of direct engagement and modulation of the host antiviral responses, highlight the constant pressures exerted by the co-evolutionary arms race between host and viral pathogens [42,43]. In fact, the high divergence observed in the new rPox-host range protein in MYXV-Tol, might suggest that the parental virus from which this recombinant region was donated is able to replicate within a completely different host species that has a unique repertoire of anti-viral response pathways. This might ultimately result in a new poxvirus capable of differentially modulating the anti-viral responses of hare cells, compared to MYXV, playing a critical role in species leaping and virus pathogenicity, in the new host. Nevertheless, the biological implications of the new genes found in the recombination “cassette”, still need to be experimentally addressed.

The data presented in this paper report that MYXV-Tol is a result of a recombinant event between a MYXV virus and a still-unreported poxvirus that shares common ancestral sequences to that of capripoxviruses, cervidpoxviruses, suipoxviruses, yatapoxviruses, and three unclassified poxviruses (BeAn 58058 virus, cotia virus, and eptesipoxvirus). Recent reports genetically characterized a large number of European and Australian strain MYXV genomic sequences [4,27] and haplotypes, which are usually suitable for tracking the spread of MYXV virus [44]. However, the discrimination of alterations in MXYV genomes that are responsible for increased virulence grades or attenuated phenotypes is still a complicated task. While the precise mechanisms that allowed the MYXV-Tol to apparently acquire virulence and leap into the Iberian hares species are not yet understood, the genetic characterization of this novel MYXV-Tol virus, in combination with further studies of the proteins found in the new recombinant insertion region, will help provide the foundation to a better understanding of this cross-species transmission.

## Supplementary Materials

The following are available online at https://www.mdpi.com/1999-4915/11/6/530/s1.
