# Supplementary material for: Genetic Characterization of a Recombinant Myxoma Virus in the Iberian Hare (Lepus granatensis)
Source: Viruses. 2019 Jun 7;11(6):530. doi: 10.3390/v11060530 (PMC6631704; doi:10.3390/v11060530)
Supplement: Supplementary file 1 [file viruses-11-00530-s001.zip › Supplementary figure 1.pdf]

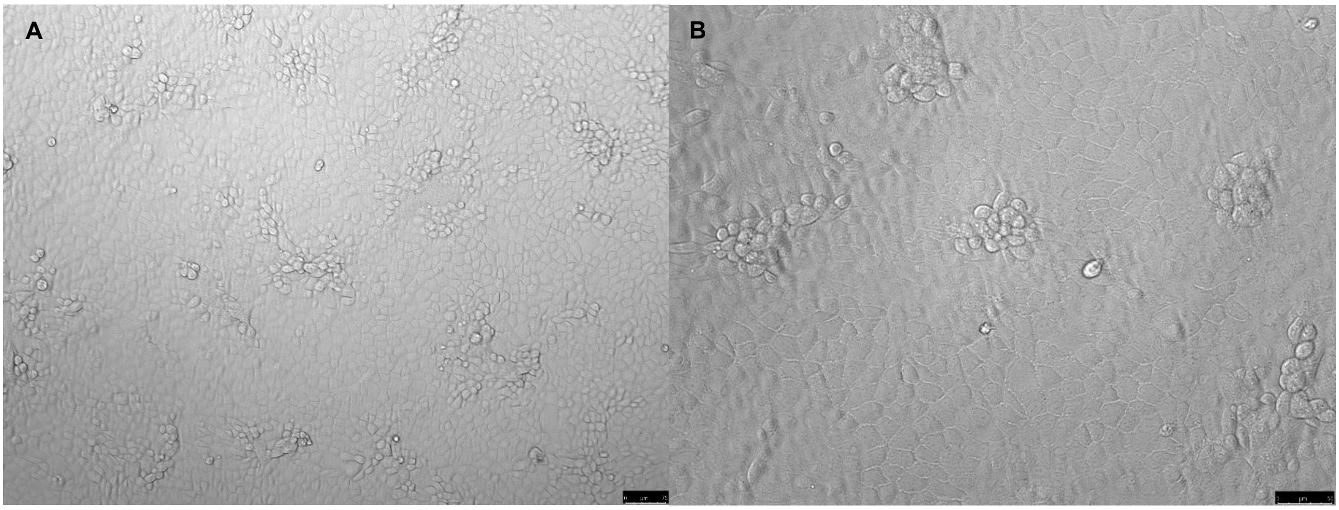

Figure S1: After performing a serial dilution of the purified MYXV-To virus, RK13 cells were infected and incubated for 48 hours at 37ºC. At 2 days post infection, a typical MYXV cyto-pathic effect (foci formation) was visualized using a Leica DMI6000 B inverted microscope at 10x (A) and 20x (B).
