# Supplementary material for: Genetic Characterization of a Recombinant Myxoma Virus in the Iberian Hare (Lepus granatensis)
Source: Viruses. 2019 Jun 7;11(6):530. doi: 10.3390/v11060530 (PMC6631704; doi:10.3390/v11060530)
Supplement: Supplementary file 1 [file viruses-11-00530-s001.zip › Supplementary figure 3.pdf]

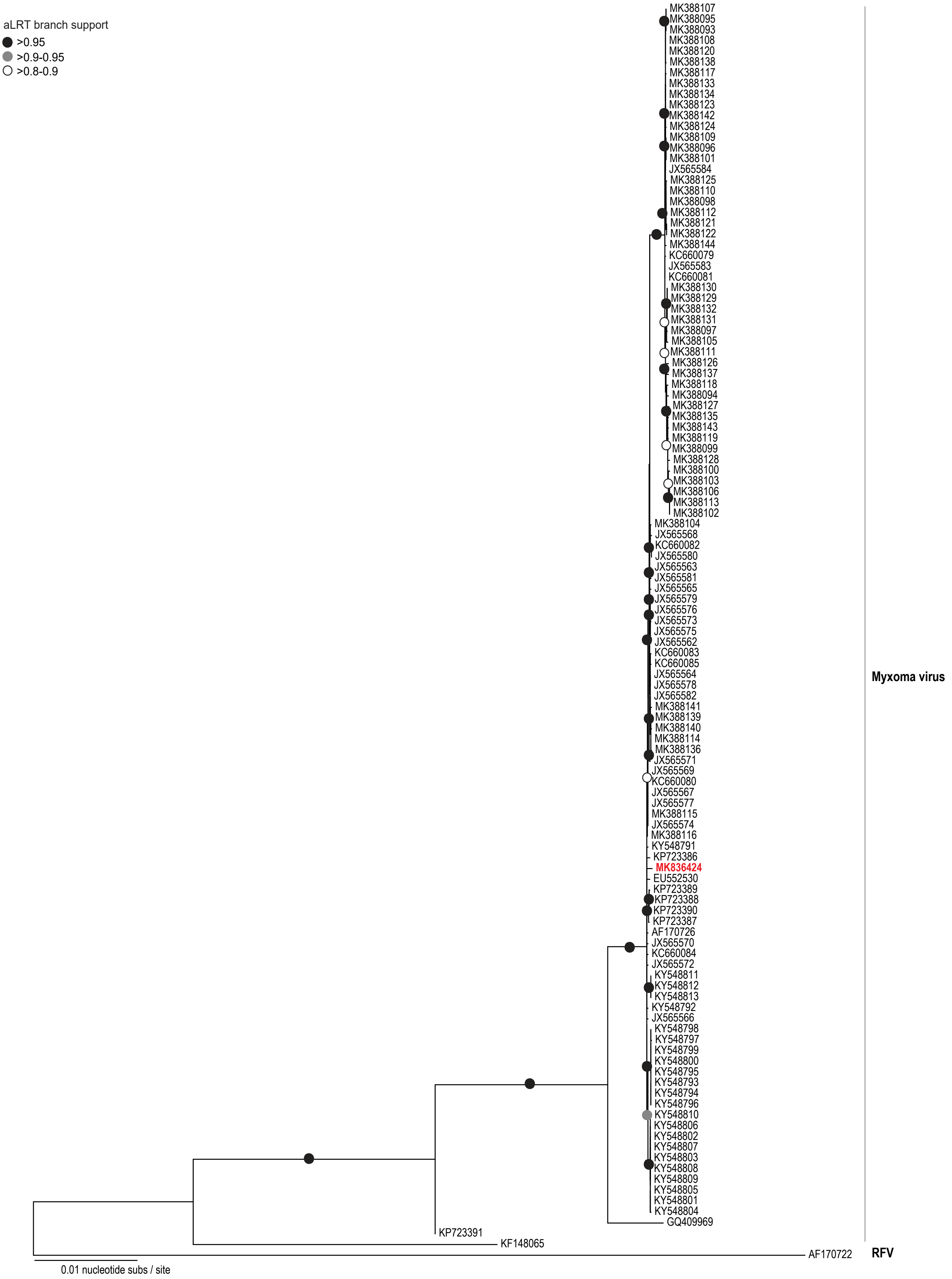

Figure S3: Maximum likelihood phylogentic tree of the aligned genomes of MYXV and RFV. Branches with aLRT support 0.95 are indicated with black circles whereas branches exhibiting 0.9-0.95 and 0.8-0.9 are indicated with grey and white circles, respectively.
