# Supplementary figures and images for: Genetic Characterization of a Recombinant Myxoma Virus in the Iberian Hare (Lepus granatensis)
Source: Viruses. 2019 Jun 7;11(6):530. doi: 10.3390/v11060530 (PMC6631704; doi:10.3390/v11060530)

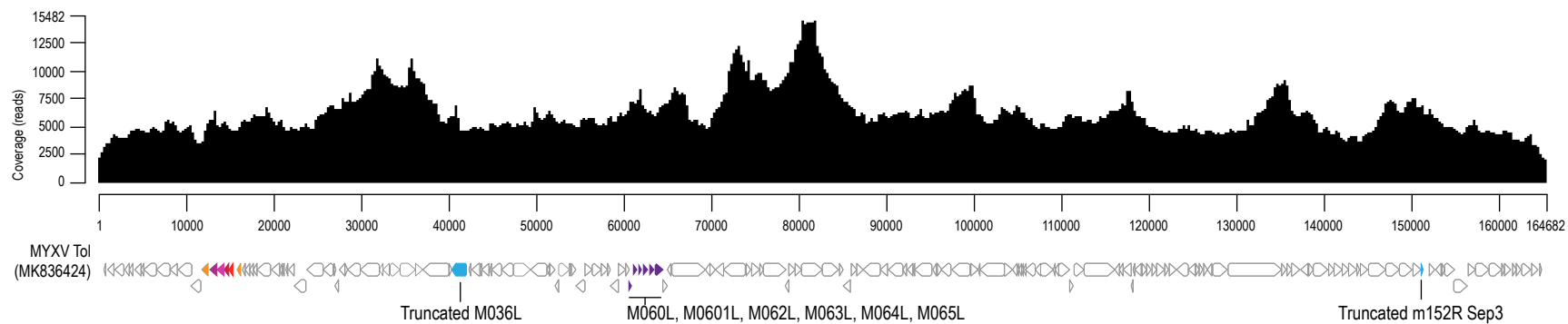

Figure S2: Posterior mapping of the Illumina sequencing read to the genome of MYXV-To using BBmap [16].

Supplement: Supplementary file 1 [file viruses-11-00530-s001.zip › Supplementary figure 2.pdf]
